# Human papillomavirus type 16 and 18 in urothelial bladder neoplasms: detection, quantification, and comparison with non-neoplastic samples

**DOI:** 10.1186/s13027-025-00725-4

**Published:** 2025-12-23

**Authors:** Arshia Yazdani, Ghodsieh Kamrani, Arefeh Ebrahimian Shiadeh, Emadoddin Moudi, Zeinab Vosough, Hoda Shirafkan, Farzin Sadeghi, Akramossadat Hosseini

**Affiliations:** 1 Student Research Committee, Babol University of Medical Sciences, Babol, Iran; 2 Department of Pathology, School of Medicine, Babol University of Medical Sciences, Babol, Iran; 3 Department of Medical Microbiology, Faculty of Medicine, Golestan University of Medical Science, Gorgan, Iran; 4 Clinical Research Development Center, Shahid Beheshti Hospital, Babol University of Medical Sciences, Babol, Iran; 5 Social Determinants of Health Research Center, Health Research Institute, Babol University of Medical Science, Babol, Iran; 6 Cellular and Molecular Biology Research Center, Health Research Institute, Babol University of Medical Sciences, Babol, Iran

**Keywords:** Bladder cancer, Human papillomavirus, Urothelial neoplasm

## Abstract

**Background:**

Bladder cancer is a prevalent urothelial cancer, and recent studies have focused on the potential role of Human Papillomavirus (HPV) in its development. This study investigates the prevalence and viral load of HPV types 16 and 18 in bladder urothelial neoplasms and compares these findings with non-neoplastic samples.

**Methods:**

A retrospective cross-sectional study was conducted using tissue samples from patients with urothelial bladder carcinoma (UBC) and a non-neoplastic control group. DNA was extracted and analyzed using real-time PCR to detect and quantify HPV types 16 and 18.

**Results:**

Although the prevalence of high-risk HPV types (16 and 18) was higher in bladder cancer patients compared to controls, the difference was not statistically significant. However, HPV-16 viral load was significantly higher in patients (*P* < 0.001), and HPV-18 viral load was also significantly elevated (*P* = 0.004). Both HPV-16 and HPV-18 viral loads were generally higher in bladder cancer patients compared to healthy individuals.

**Conclusion:**

This study demonstrated an increased prevalence of high-risk HPV types in bladder urothelial neoplasms, with elevated viral loads for HPV-16 and HPV-18 in the cancer group. While these differences were not statistically significant, the increased viral load suggests that HPV may play a role in bladder cancer progression, particularly in cases of persistent infection. These findings highlight the importance of strengthening HPV vaccination programs and further investigation into the long-term effects of HPV on bladder cancer.

## Introduction

Bladder cancer is a significant global health concern, ranked as the tenth most common cancer worldwide and a major contributor to cancer-related mortality [1]. It most commonly manifests as urothelial bladder carcinoma (UBC), which is classified into non-muscle-invasive bladder cancer (NMIBC) and muscle-invasive bladder cancer (MIBC) based on the extent of tumor invasion [2]. Although advancements in diagnostic and therapeutic strategies have been made, the prognosis for advanced bladder cancer remains poor [3], highlighting the need for further investigation into its causes and molecular mechanisms. While substantial research has been conducted on genetic and environmental factors associated with bladder cancer, the role of viral infections, particularly Human Papillomavirus (HPV), remains underexplored. HPV, a DNA virus from the Papillomaviridae family, is well known for its oncogenic potential in several cancers, including cervical, anogenital, and oropharyngeal cancers [4]. Among the high-risk HPV types, HPV-16 and HPV-18 are most frequently associated with cancer development through the expression of E6 and E7 oncoproteins, which disrupt tumor suppressor pathways involving p53 and retinoblastoma (Rb) proteins [5]. Despite some studies linking HPV infection to bladder cancer, the association remains debated, with findings often conflicting. While certain studies suggest a strong connection, others report no significant relationship, underscoring the need for further investigation to clarify this issue [6, 7]. Data on the prevalence of HPV in bladder cancer patients is limited, particularly in regions with high HPV infection rates, and findings have often been inconsistent [7–11]. This research aims to address these gaps by investigating the prevalence and viral load of HPV-16 and HPV-18 in urothelial carcinoma samples from bladder tissue, comparing these findings with non-neoplastic bladder samples. Using advanced molecular techniques, such as real-time polymerase chain reaction (PCR), the study seeks to clarify the potential contribution of high-risk HPVs to the development and progression of bladder cancer. Additionally, it will examine the relationship between HPV viral load and clinicopathological factors, such as tumor stage and grade, providing valuable insights into its prognostic and therapeutic significance. By focusing on a well-defined group of patients and employing robust methodologies, this study will contribute to the ongoing discussion about the oncogenic role of HPV in bladder cancer. Furthermore, it will offer practical implications for clinical management and help inform the development of HPV-targeted therapies, which could play a significant role in future cancer control strategies.

## Methodology

### Study design and population

This cross-sectional study aimed to investigate the prevalence and viral load of high-risk HPV types 16 and 18 in urothelial carcinoma (UBC) samples from bladder tissue, comparing them with non-neoplastic bladder tissue. The study was conducted at Shahid Beheshti Hospital in Babol, Iran, between 2019 and 2020, using archived tissue samples collected during routine clinical procedures (TURBT for cases, cystoscopy for controls). Control group participants were patients with non-neoplastic conditions requiring cystoscopy, including urinary tract stones, benign prostatic hyperplasia, or urethral stricture. All control tissues underwent histopathological examination to confirm the absence of neoplastic changes before inclusion in the study. Recruitment criteria required: (1) histologically confirmed non-neoplastic bladder tissue, (2) no history of bladder cancer or other malignancies, and (3) absence of immune-compromising conditions or immunosuppressive therapy. Inclusion criteria consisted of histologically confirmed UBC or non-neoplastic bladder conditions, while exclusion criteria included immune-compromising conditions or treatment with immunosuppressive drugs or incomplete medical records. A total of 113 samples were selected using convenience sampling, with 60 from UBC patients and 53 from the control group.

### Ethical considerations

Ethical approval was obtained from the Babol University of Medical Sciences Ethics Committee (IR.MUBABOL.HRI.REC.1402.053). Written informed consent was secured from all participants, and confidentiality was maintained by anonymizing the data.

### Data collection and laboratory protocols

After obtaining written consent, fresh tumor tissue samples were collected during Transurethral resection of bladder tumor (TURBT) from UBC patients and via cystoscopy from controls. Samples were immersed in Protector RNA solution and stored at -20 °C. DNA was extracted using a commercial kit, and its concentration and purity were assessed with a spectrophotometer. To minimize technical bias, all samples (TURBT specimens and cystoscopic biopsies) underwent identical processing: (1) immersion in Protector RNA solution immediately post-collection, (2) storage at -20 °C, (3) DNA extraction using AddPrep R Genomic DNA Extraction Kit (Addbio, South Korea), and (4) spectrophotometric quality control (DNA concentration ≥ 10 ng/µL; A260/A280 1.8-2.0). Samples failing quality control were excluded. Real-time PCR was used to detect and quantify HPV types 16, 18 and human RNase P, employing specific primers and probes [12–14]. Real-time PCR reactions included positive and negative controls for accuracy. Thermocycling conditions were as follows: initial denaturation at 95 °C for 10 min, followed by 40 cycles of denaturation at 95 °C for 15 s, annealing/extension at 60 °C for 1 min. The viral load per cell was calculated by normalizing the number of HPV copies to the copies of the RNase P gene per microliter. Given that human diploid cells contain two copies of RNase P, the formula used was: (Viral Copies per µL) / (1/2 RNase P Copies per µL) = Viral Copies per Cell. Viral quantification was validated using standard curves derived from serial dilutions of plasmid DNA containing conserved regions of the RNase P and HPV E6 genes, which confirmed the reliability of the viral load measurements. Table 1 presents the primers and probes used for the detection of the target genes RNase P and HPV E6.

**Table 1:** Primers and probes for target gene detection of RNase P and HPV E6

Target Gene	Primer/Probe	Sequences (5’-3’)	Refs
Human RNase P	RNP F	5’-AGATTTGGACCTGCGAGCG-3’	[13]
	RNP R	5’-GAGCGGCTGTCTCCACAAGT-3’	
	RNP Probe	FAM-TTCTGACCTGAAGGCTCTGCGCG-BHQ1	
HPV16-E6	E6 F-Primer	5’-GAGAACTGTAATGTTTCAGGACC-3’	[14]
	E6 R-Primer	5’-GCACAGAGCTGTAAACAACTATACA-3`	
	E6 Probe	5’- FAM-CAGGAGCGACCTAGAAAGTTACCACAGTT-BHQ1-3`	
HPV18-E6	E6 F-Primer	5’-TCACAACATAGCTGGGCACT-3`	[12]
	E6 R-Primer	5’-CTTGTGTTTCTCTGCGTCGT-3`	
	E6 Probe	5’- FAM-GCCATTCGTGCTGCAACCGA-BHQ1-3`	

### Statistical analysis

Statistical Analysis Data were analyzed with SPSS version 26. Descriptive statistics summarized demographic characteristics. Continuous variables (age, BMI, WBC, lymphocytes) were compared using independent samples t-tests after confirming normality with Shapiro-Wilk tests. Categorical variables were analyzed using chi-square tests. For categorical variables with expected cell counts < 5, Fisher’s exact test was used instead of chi-square tests. Mann-Whitney U tests were used to assess viral load differences between groups. Statistical significance was set at p-value < 0.05.

## Results

This study investigated the relationship between demographic, clinical, behavioral, and virological factors with bladder cancer. The analyses focused on differences between individuals with bladder cancer (case group) and those without (control group), as well as the role of HPV types 16 and 18 in bladder cancer.

### Demographic and clinical characteristics

This retrospective cross-sectional study included 113 individuals who presented at hospitals in Babol from 2019 to 2020. Based on the inclusion criteria, 60 patients (53.1%) diagnosed with bladder cancer constituted the case group, while 53 individuals (46.9%) without bladder cancer or a history of bladder tumors served as the control group. The mean age of participants in the control group was 61.27 ± 17.20 years, compared to 63.92 ± 12.77 years in the case group. No statistically significant difference was observed in age between the two groups (*p* = 0.364). The mean body mass index (BMI) was 27.23 ± 3.39 in the control group and 26.45 ± 4.55 in the case group, with no significant difference (*p* = 0.307). The mean white blood cell (WBC) count was significantly higher in the case group (8466.00 ± 2774.92) compared to the control group (7240.19 ± 1997.76; *p* = 0.008). The mean lymphocyte count showed no significant difference between the case group (32.33 ± 5.67) and the control group (32.77 ± 7.16; *p* = 0.716). Table 2 summarizes these findings.

**Table 2:** Comparison of demographic and clinical variables between groups

Variable	Control (*n* = 53)	Case (*n* = 60)	*P*-value†
Age (years)	61.27 ± 17.20	63.92 ± 12.77	0.364
BMI (kg/m²)	27.23 ± 3.39	26.45 ± 4.55	0.307
WBC (cells/µL)	7240.19 ± 1997.76	8466.00 ± 2774.92	0.008
Lymphocytes (%)	32.77 ± 7.16	32.33 ± 5.67	0.716

† Continuous variables analyzed with t-tests; categorical variables with chi-square tests

### Behavioral and lifestyle factors

As shown in Table 3, gender and diabetes status were not significantly associated with bladder cancer (*p* > 0.05). However, illicit use of drugs was significantly more prevalent in the case group (36.7%) than in the control group (13.2%; *p* = 0.004). Similarly, smoking was significantly more common in the case group (50.0%) than in the control group (13.2%; *p* < 0.001).

**Table 3:** Association of behavioral and lifestyle factors with bladder cancer

Variable	Control Group (%)	Case Group (%)	*p*-value
Gender (Male)	94.3	86.7	0.170
Diabetes	26.4	23.3	0.705
Illicit use of drugs	13.2	36.7	0.004
Smoking	13.2	50.0	< 0.001

### HPV-18 and HPV-16 analysis

The prevalence of HPV-18 was higher in the case group (8.3%) compared to the control group (5.7%), though this was not statistically significant (*p* = 0.580). Similarly, HPV-16 prevalence was 11.7% in the case group and 7.5% in the control group (*p* = 0.461). Table 4 illustrates the distribution of HPV-18 and HPV-16 among control and case groups, along with their statistical association.

**Table 4:** Distribution of HPV-18 and HPV-16 across control and case groups

Variable	Group	Control (*n* = 53)	Case (*n* = 60)	*P*-Value
HPV-18	Negative	50 (94.3%)	55 (91.7%)	0.573
	Positive	3 (5.7%)	5 (8.3%)	
HPV-16	Negative	49 (92.5%)	53 (88.3%)	0.456
	Positive	4 (7.5%)	7 (11.7%)	

p-values calculated using Fisher’s exact test for expected cell counts < 5

**Fig. 1:**
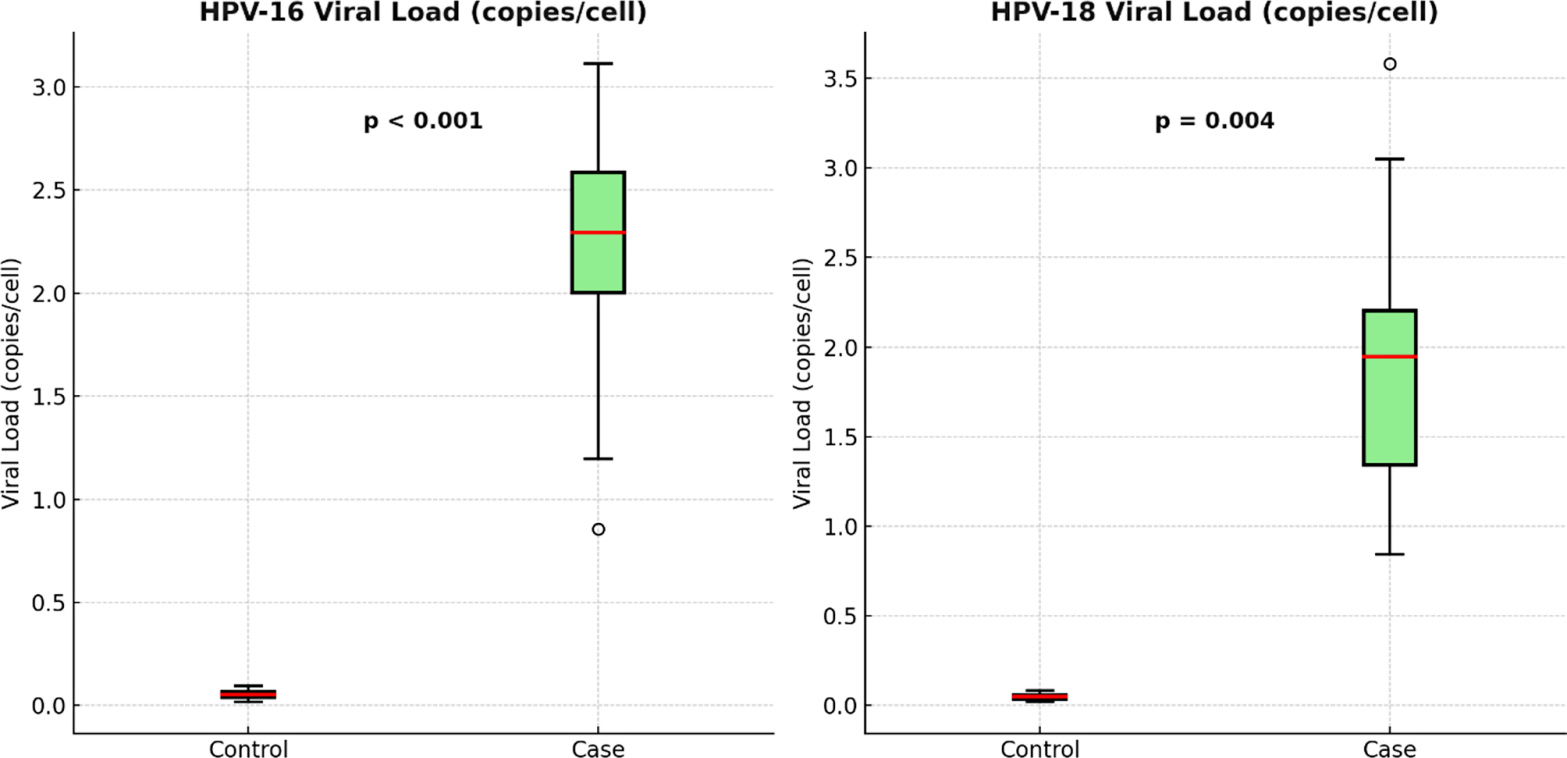
Comparison of HPV-16 and HPV-18 viral loads between bladder cancer patients and non-neoplastic controls. Boxplots illustrate significantly higher viral loads of HPV-16 (*p* < 0.001) and HPV-18 (*p* = 0.004) in the case group compared to controls. Data are expressed as viral copies per cell

As detailed in Table 5 and visually depicted in Fig. 1, significant differences were observed in viral loads between groups. The mean HPV-18 viral load was substantially higher in bladder cancer patients (1.823 ± 0.646 copies per cell) compared to controls (0.049 ± 0.014; *p* = 0.004). Similarly, HPV-16 viral loads were significantly elevated in the case group (2.269 ± 0.539 copies per cell) versus the control group (0.058 ± 0.020; *p* < 0.001).

**Table 5:** Comparison of HPV viral loads between groups

Variable	Control (*n* = 53)	Case (*n* = 60)	*P*-value
HPV-18 (copies/cell)	0.049 ± 0.014	1.823 ± 0.646	**0.004**
HPV-16 (copies/cell)	0.020 ± 0.058	2.269 ± 0.539	**< 0.001**

### Tumor characteristics and HPV analysis

Table 6 presents a comprehensive analysis of tumor characteristics, HPV prevalence, and their associations in UBC cases and controls. The majority of tumors were low-grade (58.3%), non-muscle-invasive (76.7%), and of pure urothelial histology (86.7%). The overall prevalence of high-risk HPV (HPV-16 and/or HPV-18) was 18.3% in cases versus 13.2% in controls (*p* = 0.542), with co-infection observed in 1 case (1.7%). No statistically significant associations were found between HPV status and tumor characteristics (*p* > 0.05 for all). Notably, among the 14 T2 + tumors, one case was positive for HPV-16 and one for HPV-18 (totaling 2 cases, 14.3%), though this was not statistically significant (*p* = 0.312).

**Table 6:** Association between HPV subtypes and clinicopathological features in bladder carcinoma

Variable	Category	Total (*n*)	HPV-16 + *n*(%)	HPV-18 + *n*(%)	^†^Any HPV + *n*(%)	*P*-value
Tumor stage	Ta	28	4 (14.3%)	3 (10.7%)	6 (21.4%)	0.312
	T1	18	2 (11.1%)	1 (5.6%)	3 (16.7%)	
	T2+	14	1 (7.1%)	1 (7.1%)	2 (14.3%)	
Tumor grade	Low-grade	35	3 (8.6%)	2 (5.7%)	5 (14.3%)	0.999
	High-grade	25	4 (16.0%)	3 (12.0%)	7 (28.0%)	
Histologic type	Pure urothelial	52	6 (11.5%)	4 (7.7%)	9 (17.3%)	0.999
	Variant types	8	1 (12.5%)	1 (12.5%)	2 (25%)	
Overall HPV prevalence	Cases (*n* = 60)	–	7 (11.7%)	5 (8.3%)	11 (18.3%)	0.542*
	Controls (*n* = 53)	–	4 (7.5%)	3 (5.7%)	7 (13.2%)	
Co-infection	Cases	–	1 (1.7%)	1 (1.7%)	–	0.467*
	Controls	–	0 (0%)	0 (0%)	–	

† The “Any HPV+” column represents the total number of samples positive for at least one HPV type, calculated as: (HPV-16 + cases) + (HPV-18 + cases) - (Co-infection cases within that category).*p-values calculated using Fisher’s exact test

### Subgroup analyses

No significant associations were found between HPV status and sex, age group (< 70 years vs. ≥70 years), disease status or diabetes status (*p* > 0.05). However, there was a significant association between smoking and HPV-16 prevalence (*p* = 0.042), with smokers exhibiting an 18.9% prevalence compared to 5.3% in non-smokers. Similarly, HPV-18 prevalence was higher among illicit drug users (17.2%) compared to non-users (3.6%, *p* = 0.031). Although the prevalence of overall HPV infection (HPV-16 and/or HPV-18) appeared higher in patients with relapsed disease (21.1%) compared to those with new diagnoses (7.3%), this difference did not achieve statistical significance (*p* = 0.189, Fisher’s exact test). Table 7 presents the association of demographic and clinical variables with HPV-16 and HPV-18 status.

**Table 7:** Association of demographic and clinical variables with HPV-16 and HPV-18 status

Variables			HPV-16 n (%)		P-value	HPV-18 n (%)		P-value
		Total	Negative (*n* = 102)	Positive (*n* = 11)		Negative (*n* = 105)	Positive (*n* = 8)	
Age (years)	**≤ 70**	69	61 (88.4)	8 (11.6)	0.592	67 (97.1)	2 (2.9)	0.853
	**> 70**	44	41 (93.2)	3 (6.8)		38 (86.4)	6 (13.6)	
Gender	Male	102	93 (91.2)	9 (8.8)	0.320	95 (93.1)	7 (6.9)	0.784
	Female	11	9 (81.8)	2 (18.2)		10 (90.9)	1 (9.1)	
Disease Status	Control	53	49 (92.5)	4 (7.5)	0.189	50 (94.3)	3 (5.7)	0.265
	New	41	38 (92.7)	3 (7.3)		39 (95.1)	2 (4.9)	
	Relapse	19	15 (78.9)	4 (21.1)		16 (84.2)	3(15.8)	
Diabetes	No	85	77 (90.6)	8 (9.4)	0.84	80 (94.1)	5 (5.9)	0.387
	Yes	28	25 (89.3)	3 (10.7)		25 (89.3)	3 (10.7)	
illicit use of drugs	No	84	76 (90.5)	8 (9.5)	0.898	81 (96.4)	3 (3.6)	*p* = 0.031
	Yes	29	26 (89.7)	3 (10.3)		24 (82.8)	5(17.2)	
Smoking	No	76	72 (94.7)	4 (5.3)	*p* = 0.042	71(93.4)	5 (6.6)	0.766
	Yes	37	30 (81.1)	7 (18.9)		34 (91.9)	3 (8.1)	

Chi-square test was used. Significant values are highlighted in bold. p-values calculated using Fisher’s exact test for expected cell counts < 5

### Multivariate logistic regression analysis

Logistic regression revealed that smoking increased the odds of bladder cancer by 8.050 times (95% CI: 2.514–25.733, *p* < 0.001). Other variables, including BMI, diabetes, and HPV-18/HPV-16, were not significant predictors of bladder cancer (*p* > 0.05).

### Disease grade and stage

There was no statistically significant association between HPV-18 or HPV-16 positivity and tumor grade or stage in the case group (*p* > 0.05). However, patients with higher-grade tumors had higher average HPV-16 viral loads (3.258 copies per cell) compared to those with low-grade tumors (2.104 copies per cell), but the difference was not statistically significant (*p* = 0.404).

## Discussion

This study aimed to examine the relationship between high-risk HPV types 16 and 18 and bladder cancer, specifically focusing on viral load in patients with urothelial carcinoma of the bladder and a non-cancerous control group. Our results demonstrated a higher prevalence of high-risk HPV types 16 and 18 in the bladder cancer group compared to the control group. However, despite these findings, no statistically significant correlation was found between the presence of HPV and bladder cancer. Notably, the study revealed significant differences in viral load between the bladder cancer patients and the control group, with the cancer patients exhibiting a notably higher viral load. The elevated viral load in the bladder cancer patients suggests an association between HPV infection (particularly at higher quantities) and bladder cancer, though causality remains unproven. Higher viral loads may indicate active viral replication in the affected tissue and persistent HPV infection in individuals [15]. Additionally, higher viral loads are generally associated with a higher risk of cellular changes and carcinogenesis, as they increase the production of viral oncogenes such as E6 and E7, which can disrupt cellular functions like DNA repair and cell cycle control. This may lead to increased tumor proliferation, reduced apoptosis, and eventually more aggressive cancers [16]. Furthermore, persistent HPV infection, especially when combined with other risk factors such as smoking or chronic inflammation, may act as a catalyst for tumor development or intensify existing cancer [17, 18]. While the study did not establish a direct causal relationship between HPV and bladder cancer, the higher viral load could indicate a more chronic or severe infection, which might contribute to tumorigenesis. Although HPV infection alone may not be sufficient to cause bladder cancer, its presence, especially in combination with other environmental and lifestyle factors, could facilitate malignancy development. The results of this study, thus, align with the hypothesis that HPV may act as an important co-factor in bladder cancer development, though its role remains inconclusive. These findings are consistent with prior research that has explored the potential link between HPV and bladder cancer. For instance, systematic reviews and meta-analyses have shown variable results regarding HPV’s role in bladder cancer, with some studies suggesting a potential association and others indicating no significant correlation [7–9]. Similar to our findings, studies by Khatami et al. and Polesel et al. reported high HPV prevalence in bladder cancer patients, but no direct statistical link was established [9, 19]. Conversely, other studies such as those by Eslami et al. and Moghadam Ohadian et al. found a more substantial relationship between HPV infection and bladder cancer, particularly concerning tumor grade and recurrence [6, 20]. The discrepancies between studies may stem from factors such as variations in sample sizes, methodologies, and geographic or population-specific characteristics. Differences in diagnostic methods, including PCR and hybridization techniques, could also explain the observed variations. Additionally, regional factors such as smoking rates can influence bladder cancer risk. For instance, populations in areas with high smoking prevalence may have a higher risk due to the combined effects of smoking and HPV infection [21]. Understanding these geographic variations is essential for developing tailored prevention and treatment strategies. Moreover, regions with high HPV vaccination rates tend to report lower HPV-related cancer prevalence, which could alter the observed viral load in bladder cancer patients [22]. The findings of this study highlight the potential role of HPV as a biomarker for bladder cancer progression and recurrence. This underscores the importance of HPV vaccination programs, particularly in regions with high smoking rates, as a preventive strategy [10]. Given the chronic nature of HPV infection and its possible contribution to tumorigenesis, vaccination efforts targeting high-risk HPV strains could help mitigate the burden of HPV-related cancers, including bladder cancer [19]. Although no statistically significant association was found between HPV status and disease relapse (*p* = 0.189), the observed trend of higher HPV prevalence in relapsed cases (21.1% vs. 7.3%) is noteworthy and suggests a potential role for HPV in bladder cancer recurrence. Plausible hypotheses include the impact of chronic HPV infection through sustained E6/E7 expression, induction of field cancerization in the urothelium, or differential treatment responses. However, this finding must be interpreted with caution due to study limitations (small sample size, cross-sectional design). Larger, prospective longitudinal studies are needed to further investigate this relationship and its implications for therapeutic and preventive strategies. While this study provides valuable insights into HPV’s role in bladder cancer, several limitations should be considered. The small sample size and single geographic location limit the generalizability of the findings. While standardized protocols were applied, differences in tissue quantity and cellular composition between TURBT specimens (cases) and cystoscopic biopsies (controls) represent a potential limitation. Although HPV detection was normalized to RNase P copies, variations in tumor cellularity or stromal content could theoretically impact DNA yield and assay sensitivity. This highlights the importance of validating our findings in larger cohorts with prospectively matched tissue samples. Additionally, the lack of longitudinal follow-up prevents an analysis of long-term HPV effects. PCR detection, while effective, may not capture all HPV infections, especially in samples with low viral loads. Furthermore, this study was limited to the detection and quantification of HPV types 16 and 18, and did not investigate other high-risk HPV subtypes that have been implicated in bladder carcinogenesis. Future studies should include broader HPV genotyping to provide a more comprehensive understanding of the viral etiology in bladder cancer and investigate the potential association between HPV infection and tumor recurrence or progression. Large-scale, longitudinal studies are needed to clarify the relationship between HPV and bladder cancer and to explore the interaction with other risk factors, such as smoking and environmental carcinogens. Investigating HPV’s role in recurrence and progression may also lead to new therapeutic avenues. The elevated viral load observed in patients with chronic or severe HPV infections suggests that targeted HPV vaccination programs could serve as an effective preventive strategy, particularly in regions with high smoking rates. Understanding HPV’s role in cancer progression may also contribute to the development of HPV-targeted therapies, improving treatment outcomes and reducing recurrence.

In conclusion, while this study did not establish a direct causal relationship between HPV and bladder cancer, the observed increase in viral load among bladder cancer patients suggests a potential association between HPV infection and bladder cancer, particularly in cases of chronic or severe infection. These findings call for further investigation into the mechanisms by which HPV may contribute to bladder cancer progression and recurrence. Ultimately, understanding HPV’s role in bladder cancer could inform preventative strategies, such as vaccination, and contribute to the development of more targeted treatments.

## Data Availability

The datasets used and/or analyzed during the current study are available from the corresponding author on reasonable request.

## References

[1] Zhang Y, Rumgay H, Li M, Yu H, Pan H, Ni J. The global landscape of bladder cancer incidence and mortality in 2020 and projections to 2040. J Global Health. 2023;13. 10.7189/jogh.13.04109 PMC10502766 37712386

[2] Schwarzova L, Varchulova Novakova Z, Danisovic L, Ziaran S. Molecular classification of urothelial bladder carcinoma. Mol Biol Rep. 2023;50(9):7867–77. 37525073 10.1007/s11033-023-08689-7 PMC10460735

[3] Patel VG, Oh WK, Galsky MD. Treatment of muscle-invasive and advanced bladder cancer in 2020. Cancer J Clin. 2020;70(5):404–23. 10.3322/caac.21631 32767764

[4] Berman TA, Schiller JT. Human papillomavirus in cervical cancer and oropharyngeal cancer: one cause, two diseases. Cancer. 2017;123(12):2219–29. 28346680 10.1002/cncr.30588

[5] Basukala O, Banks L. The not-so-good, the bad and the ugly: HPV E5, E6 and E7 oncoproteins in the orchestration of carcinogenesis. Viruses. 2021;13(10):1892. 34696321 10.3390/v13101892 PMC8541208

[6] Ohadian Moghadam S, Mansori K, Nowroozi MR, Afshar D, Abbasi B, Nowroozi A. Association of human papilloma virus (HPV) infection with oncological outcomes in urothelial bladder cancer. Infect Agents Cancer. 2020;15:1–8. 10.1186/s13027-020-00318-3 PMC7456036 32874199

[7] Sun JX, Xu JZ, Liu CQ, An Y, Xu MY, Zhong XY, et al. The association between human papillomavirus and bladder cancer: evidence from meta-analysis and two‐sample Mendelian randomization. J Med Virol. 2023;95(1):e28208. 36226344 10.1002/jmv.28208 PMC10092419

[8] Dolgasheva DS, Ibragimova MK, Tsyganov MM, Litviakov NV. Human papillomavirus and bladder cancer: literature review and meta-analysis. Afr J Urol. 2024;30(1):11.

[9] Khatami A, Salavatiha Z, Razizadeh MH. Bladder cancer and human papillomavirus association: a systematic review and meta-analysis. Infect Agents Cancer. 2022;17(1):3. 10.1186/s13027-022-00415-5 PMC8780707 35062986

[10] Muresu N, Di Lorenzo B, Saderi L, Sechi I, Del Rio A, Piana A, Sotgiu G. Prevalence of human papilloma virus infection in bladder cancer: a systematic review. Diagnostics. 2022;12(7):1759. 35885662 10.3390/diagnostics12071759 PMC9318826

[11] Yao X, Xu Z, Duan C, Zhang Y, Wu X, Wu H, et al. Role of human papillomavirus and associated viruses in bladder cancer: an updated review. J Med Virol. 2023;95(9):e29088. 37706751 10.1002/jmv.29088

[12] Damay A, Didelot-Rousseau MN, Costes V, Konate I, Ouedraogo A, Nagot N, et al. Viral load and physical status of human papillomavirus (HPV) 18 in cervical samples from female sex workers infected with HPV 18 in Burkina Faso. J Med Virol. 2009;81(10):1786–91. 19697418 10.1002/jmv.21554

[13] Imajoh M, Hashida Y, Taniguchi A, Kamioka M, Daibata M. Novel human polyomaviruses, Merkel cell polyomavirus and human polyomavirus 9, in Japanese chronic lymphocytic leukemia cases. J Hematol Oncol. 2012;5(1):25. 22658224 10.1186/1756-8722-5-25 PMC3407023

[14] Peitsaro P, Johansson B, Syrjänen S. Integrated human papillomavirus type 16 is frequently found in cervical cancer precursors as demonstrated by a novel quantitative real-time PCR technique. J Clin Microbiol. 2002;40(3):886–91. 11880410 10.1128/JCM.40.3.886-891.2002 PMC120275

[15] Das S, Kundu M, Jena BC, Mandal M. Causes of cancer: physical, chemical, biological carcinogens, and viruses. In: Biomaterials for 3D tumor modeling. Elsevier; 2020. p. 607–41.

[16] Fobian SF, Mei X, Crezee J, Snoek BC, Steenbergen RD, Hu J, et al. Increased human papillomavirus viral load is correlated to higher severity of cervical disease and poorer clinical outcome: A systematic review. J Med Virol. 2024;96(6):e29741. 38922964 10.1002/jmv.29741

[17] Fishbein A, Hammock BD, Serhan CN, Panigrahy D, Carcinogenesis. Fail Resolution inflammation? Pharmacol Ther. 2021;218:107670. 10.1016/j.pharmthera.2020.107670 PMC7470770 32891711

[18] Schiffman M, Castle PE. Human papillomavirus: epidemiology and public health. Arch Pathol Lab Med. 2003;127(8):930–4. 12873163 10.5858/2003-127-930-HPEAPH

[19] Polesel J, Gheit T, Talamini R, Shahzad N, Lenardon O, Sylla B, et al. Urinary human polyomavirus and papillomavirus infection and bladder cancer risk. Br J Cancer. 2012;106(1):222–6. 22116302 10.1038/bjc.2011.519 PMC3251864

[20] Eslami G, Golshani M, Rakhshon M, Fallah F, Goudarzi H. The study on relation of human papillomavirus with bladder transitional cell carcinoma. Cancer Therapy. 2008;6:355–60.

[21] Rink M, Crivelli JJ, Shariat SF, Chun FK, Messing EM, Soloway MS. Smoking and bladder cancer: a systematic review of risk and outcomes. Eur Urol Focus. 2015;1(1):17–27. 28723350 10.1016/j.euf.2014.11.001

[22] Hirth J. Disparities in HPV vaccination rates and HPV prevalence in the united states: a review of the literature. Hum Vaccines Immunotherapeutics. 2019;15(1):146–55. 10.1080/21645515.2018.1512453 PMC6363146 30148974

